# Perovskite—a Perfect Top Cell for Tandem Devices to Break the S–Q Limit

**DOI:** 10.1002/advs.201801704

**Published:** 2019-01-30

**Authors:** Ziyu Wang, Zhaoning Song, Yanfa Yan, Shengzhong (Frank) Liu, Dong Yang

**Affiliations:** ^1^ Dalian National Laboratory for Clean Energy iChEM Dalian Institute of Chemical Physics Chinese Academy of Sciences 457 Zhongshan Road Dalian 116023 China; ^2^ University of Chinese Academy of Sciences Beijing 100049 China; ^3^ Department of Physics and Astronomy and Wright Center for Photovoltaics Innovation and Commercialization University of Toledo Toledo OH 43606 USA; ^4^ Key Laboratory of Applied Surface and Colloid Chemistry Ministry of Education Shaanxi Engineering Lab for Advanced Energy Technology School of Materials Science and Engineering Shaanxi Normal University Xi'an 710119 China; ^5^ Materials Science and Engineering Penn State University Park PA 16802 USA

**Keywords:** four‐terminal, multijunction solar cells, perovskites, portable devices, two‐terminal

## Abstract

Up to now, multijunction cell design is the only successful way demonstrated to overcome the Shockley–Quiesser limit for single solar cells. Perovskite materials have been attracting ever‐increasing attention owing to their large absorption coefficient, tunable bandgap, low cost, and easy fabrication process. With their rapidly increased power conversion efficiency, organic–inorganic metal halide perovskite‐based solar cells have demonstrated themselves as the most promising candidates for next‐generation photovoltaic applications. In fact, it is a dream come true for researchers to finally find a perfect top‐cell candidate in tandem device design in commercially developed solar cells like single‐crystalline silicon and CIGS cells used as the bottom component cells. Here, the recent progress of multijunction solar cells is reviewed, including perovskite/silicon, perovskite/CIGS, perovskite/perovskite, and perovskite/polymer multijunction cells. In addition, some perspectives on using these solar cells in emerging markets such as in portable devices, Internet of Things, etc., as well as an outlook for perovskite‐based multijunction solar cells are discussed.

## 1 Introduction

To date, the development of humankind has been predominantly driven by the use of traditional energy resources including coal, oil, and natural gas. Unfortunately, their combustion produces large amounts of oxides and suboxides of carbon, sulfur, nitrogen, etc. While only a small portion of these oxides are toxic, each of them may cause detrimental climate change and environmental pollution. It is imperative to develop and expand the usage of clean energy from renewable resources like the sun, wind, hydro, etc. Converting sunlight to electrical energy has been recognizedas the most promising approach to resolve the crises not only because it is clean but also because the sun constantly supplies far more power than we need. During the last few years, the cost of a photovoltaic (PV) system has dramatically decreased due to the rapidly increased installation volume and ever decreasing material cost, making photovoltaics not only clean but also nearly economically competitive with conventional, polluting energy resources. The most effective method to further reduce the PV cost is to improve the power conversion efficiency of the solar cells. Crystalline silicon (c‐Si)‐based solar cells are currently the dominant product in the PV market. While its record cell efficiency has reached as high as 26.6%,^1^ it is very close to the theoretical limit of 29.4% for c‐Si‐based single‐junction cell technology,^2^ and there is very little room to improve the cell efficiency.

The main component of a solar cell is a semiconductor material that can absorb photons with energies larger than its bandgap. The excess photon energy over the bandgap is not only lost by thermalization when excited electrons relax to the bottom of the conduction band, it also causes harmful heating to the system. Moreover, the photons with sub‐bandgap energies are also absorbed by the system, generating undesired heat. The trade‐off between harvesting more photons and minimizing the thermalization loss limits the theoretical maximum power conversion efficiency of the solar cells.^3^ These two losses cause the maximum possible power conversion efficiency (PCE) of a single‐junction solar cell to lie below the Shockley‐Queisser limit.^4^ To overcome the limit, several new physical concepts have been proposed, such as multijunction solar cells,^5^, ^6^, ^7^ hot carrier solar cells,^8^, ^9^, ^10^ multiple exciton generation,^11^, ^12^, ^13^ and intermediate‐band solar cells.^14^, ^15^, ^16^ However, among these concepts, only multijunction cell design has successfully been proven to overcome the Shockley–Quiesser (S–Q) limit for solar cells. For example, in the GaAs/GaInP/Ge triple junction solar cell, three component cells are used to achieve solar cell efficiency over 40% under concentrated sunlight, with each component cell using a different absorber material with a different bandgap to harvest a different wavelength range of light.^17^ Likewise, a‐Si:H/a‐SiGe:H/nc‐Si:H triple junction thin film cells have been successfully demonstrated, with the a‐Si:H top cell absorbing mainly blue light with wavelengths ranging from 300 to 550 nm, the a‐SiGe middle cell harvesting intermediate wavelength light from 550 to 700 nm, and the nc‐Si:H bottom cell converting primarily near‐infrared light with wavelengths up to 1200 nm.^18^, ^19^, ^20^, ^21^, ^22^ By doing so, the highest‐energy photons are harvested by the higher bandgap top cell absorber layer to generate a higher open‐circuit voltage (*V*_oc_). At the same time, the thermalization loss is minimized. Meanwhile, the lower‐energy photons that are transmitted through the top layers are captured by the lower bandgap bottom‐cell absorber layer to attain a broader spectrum of light utilization. Thus the multijunction devices may boost the efficiency beyond the Shockley–Queisser limit.^4^

The high‐efficient III–V solar cells have been successfully designed into multijunction cells. However, the epitaxial growth method, an advanced technology for obtaining high quality multilayers, is strict with substrate and the film grows very slow. These characters make III–V solar cell very expensive and limit the terrestrial application of III–V solar cell. There are two main issues that hinder the development of multijunction cells. One is the semiconductor material with suitable bandgap and high *V*_oc_. The other is the fabricating process of the top cell, which should be mild to the existing subcell. These issues make it very difficult to fabricate multijunction cells. Thanks to the finding of perovskite, an emerging material that has been successfully used in photovaltaic applications, multijunction cells have made a great progress in the recent years.

The hybrid organic–inorganic perovskite (HOIP) solar cell has been one of the most fascinating emerging PV technologies due to its high efficiency, low cost, ease in fabrication, tunable bandgap, high absorption coefficient and long charge carrier diffusion length.^23^, ^24^, ^25^, ^26^, ^27^, ^28^ These unique features have resulted in the conversion efficiency of perovskite solar cells increasing from 3.8% to over 22.7% in just a few years.^29^, ^30^, ^31^, ^32^, ^33^, ^34^, ^35^, ^36^ Many studies have revealed that the bandgap of perovskite can be tuned in the range from 1.17 to 2.24 eV through compositional engineering.^23^, ^37^, ^38^, ^39^, ^40^, ^41^, ^42^, ^43^ Thus, perovskite solar cells have been seen as the most promising top cells for multijunction devices.^44^, ^45^, ^46^, ^47^, ^48^, ^49^, ^50^ In addition, owing to the softness of these materials and their amazing tolerance to defects, HOIP films can be fabricated on various substrates without any need for lattice matching.^51^ These advantages make the perovskite cells ideal top cells for integration with other efficient lower‐bandgap component cells, such as single crystalline silicon cells,^45^, ^47^, ^52^, ^53^, ^54^, ^55^ CIGS cells,^48^, ^56^, ^57^, ^58^, ^59^ polymer cells,^60^, ^61^, ^62^ and lower bandgap perovskite cells.^41^, ^46^, ^63^, ^64^

## 2 Architectures and Efficiency Limits for Multijunction Solar Cells

**Figure 1:**
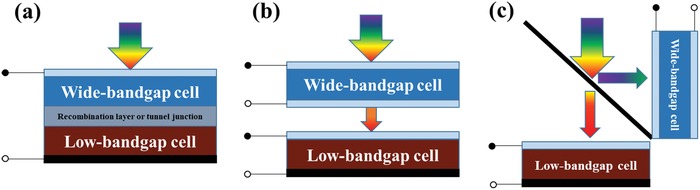
Illustration of the multijunction architectures. a) 2T monolithically integrated multijunction device. b) 4T mechanically stacked multijunction device. c) 4T optically coupled multijunction cell.

In general, there are three representative categories two‐junction tandem solar cells: two‐terminal (2T) monolithically integrated, four‐terminal (4T) mechanically stacked, and 4T optically coupled multijunction cells. **Figure** **1**a illustrates a 2T structure with the top and bottom component cells connected in series through an intermediate recombination layer. The current flowing through the series‐integrated device is governed by the Kirchhoff's law, i.e., photocurrent is limited by the smaller value of the two subcells.^65^ Therefore, the bandgap and thickness of each subcell should be precisely managed to achieve photocurrent match at the maximum power point. Because the two subcells are connected in series, the overall *V*_oc_ of the integrated cell is the sum of the *V*_oc_ of the two subcells minus the voltage loss in the tunnel junction. As it is integrated, it needs only one top transparent electrode, which is advantageous for lowering the manufacturing cost and associated parasitic absorption losses. Unfortunately, the integration process for the fabrication of 2T multijunction cells is very complicated. Because the bottom cell serves as the substrate for the growth of the top cell, its surface conditions directly affect the deposition of the top cell. Moreover, the top cell has to be fabricated without damaging the existing bottom cell.

The most challenging issue in the 2T architecture is the charge recombination layer between the two subcells. It should have low electrical resistance as well as good transparency in the near‐infrared region to guarantee that the long wavelength photons can reach the bottom cell. Meanwhile, the deposition of the recombination layers should be compatible with the underlying photovoltaic devices. Therefore, the 4T structure with a mechanically stacked top cell and bottom cell (Figure 1b) has been developed. The component cells are electrically independent due to the physical separation of the two subcells. The total PCE is the sum of those of the two subcells. There are three transparent electrodes in a 4T multijunction solar cell. The front electrode of the top cell should be able to transmit the full spectrum of photons, while the back electrode of the top cell and the front side electrode of the bottom cell should be transparent to photons in the near‐infrared region. Generally, the as‐deposited transparent electrodes of the semi‐transparent cell have little transparency compare to the transparent conducting oxide (TCO)/glass substrate. Thus, the semi‐transparent cells show higher PCE when photons are incident from the glass side.

There are also some reported multijunction solar cells based on the optically coupled 4T structure (Figure 1c). The two subcells are optically coupled by the optical splitter, which contains a dichroic mirror that splits sunlight by reflecting short wavelength photons and transmitting long wavelength photons. By distributing the split incident sunlight to each subcell, the multijunction cell can absorb the maximum portion of the incident sunlight. However, the dichroic mirrors are too expensive for large‐scale manufacturing, and the devices may be feasible only in highly concentrated PV systems, where the incident light can be focused onto a small area.^66^

**Figure 2:**
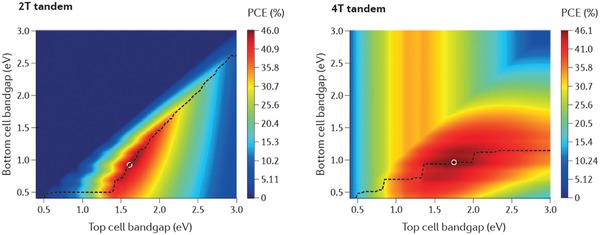
Theoretical maximum PCE for 2T and 4T tandem solar cells. Reproduced with permission.^3^ Copyright 2017, Spring Nature.

The theoretical maximum PCEs for 2T and 4T tandem solar cells as a function of the bandgaps of the top and bottom cells can be evaluated using the detailed balance principle.^3^, ^67^, ^68^ For instance, in **Figure** **2**, Eperon et al. show the theoretical maximum efficiency contour maps for tandem cells in the 2T and 4T configurations, with the dashed lines depicting the peak efficiency for a wide range of top cell bandgaps and the dots showing the global maximum PCEs.^3^ The highest PCEs for both tandem configurations can be over 46%, remarkably higher than the S‐Q limit of ≈33% for single junction cell under 1 sun illumination.^4^ Both 2T and 4T tandem configurations show board contours with PCE exceeding 33%, affording great flexibility for the choice of bandgap the top cell and corresponding bottom cell. For the 2T configuration, due to the current match requirement, the top cell bandgap for high PCE devices is stringently limited to the range of 1.5–1.9 eV. For the 4T tandem configuration, the subcells are not constrained by the current match conditions and can operate separately at their own maximum power points, resulting in a much wider selection window than its 2T counterpart. Because of the tunable bandgaps in the range of 1.5–2.2 eV, lead halide perovskites are ideal candidates for the top cell to pair with a lower bandgap cell in both 2T and 4T tandem configurations to achieve high PCEs.

## 3 Current Record Perovskite‐Based Multijunction Devices

In a general single junction perovskite solar cell, the multilayers are sequentially deposited onto the TCO‐coated glass, and finally a thick metal layer is evaporated on top of the cell. Photons are incident from the TCO/glass side and are absorbed by the perovskite layers, while the back metal contact serves as a mirror to reflect unabsorbed photons, thus ensuring that a thin perovskite layer will absorb most of the incident photons. However, in the multijunction cells, an opaque metal rear contact should be replaced by transparent conductive materials, so that photons not absorbed by the top cell can be transmitted to the bottom cell. To fulfill the requirements of superior conductivity and high transmittance, many conductive materials for the transparent electrodes have been investigated. Silver nanowires (Ag NWs) have been a promising candidate for the transparent electrode of perovskite solar cells due to their excellent transmittance in the visible and near infrared regions, and they can be deposited directly by spray coating or indirectly by mechanical transfer from flexible polyethylene terephthalate (PET).^69^, ^70^, ^71^ However, research has indicated that silver causes poor stability in perovskite cells because it can chemically react with the halogens in perovskite and produce insulating material, thus degrading the cell performance.^72^, ^73^ In addition, mechanical transfer has low reproducibility, which may limit the application of Ag NWs in large area perovskite cells. Therefore, no effort should be spared to solve these stability and reproducibility problems. The commonly used transparent electrodes are TCOs, including fluorine‐doped tin oxide (FTO),^10^ indium tin oxide (ITO),^43^, ^44^, ^45^, ^72^ aluminum‐doped ZnO (AZO),^48^, ^74^ indium zinc oxide (IZO)^75^ and hydrogenated indium oxide (IO:H).^48^, ^76^, ^77^, ^78^ These TCOs have relatively high transmittance, while the resistance is slightly higher when the films are too thin. A metal grid or fingers could enhance the conductivity with a slight decline in transmittance due to shadowing.^47^, ^79^ Electrodes based on graphene show excellent transmittance of 97% with a sheet resistance of 100 Ω sq^−1^ 80, 81 and are thus promising as transparent electrodes for perovskite solar cells. Ultrathin metal layers have also been used as the transparent electrode since they are transparent when deposited with a thickness on the scale of several nanometers.^82^, ^83^

### 3.1 Perovskite–Silicon Multijunction Solar Cells

Bailie and co‐workers demonstrated the first perovskite‐on‐silicon 4T multijunction solar cell with a PCE of 17.9% in 2014.^70^ They used Ag NWs as the transparent contact, which was mechanically transferred from flexible polyethylene terephthalate (PET) onto the top of 2,2′,7,7′‐Tetrakis (*N*,*N*‐di‐4‐methoxyphenylamino)‐9,9′‐spirobifluorene (spiro‐OMeTAD) without the use of solvent, thus avoiding damage to the underlying layers. The Ag NW film exhibits 87–90% transmission between 530 and 1000 nm. They obtained a 12.7%‐efficient semitransparent device with a transmittance of 59–77% in the range of 800–1200 nm. However, the use of sliver electrodes has been proven to affect the stability of perovskite devices through the formation of silver iodide,^73^ which may quickly degrade cell performance. TCOs deposited by sputtering have also been investigated as the transparent electrode for semitransparent perovskite solar cells. MoO*_x_* and WO*_x_* are utilized as buffer layers to protect the underlying layers for TCO deposition.^48^ Löper and co‐workers employed ITO as the transparent contact, which was deposited on a MoO_3_ buffer layer to avoid damage to the underlying layers during the sputtering process.^44^ The overall efficiency of the 4T multijunction solar cell was 13.4%. The semitransparent perovskite top cell demonstrated an efficiency of 6.2%, in contrast to an 11.6%‐efficient opaque single junction cell with a MoO*_x_*/Ag electrode. The difference was mainly caused by the enhanced sheet resistance and low reflectance of the ITO transparent electrode. Considering that the optical losses in a multijunction device are mainly caused by the parasitic absorption of the transparent contacts, the thickness of the layer should be reduced. However, the reduced thickness reduces the conductivity and leads to voltage loss and FF loss in the device. Hence, the balance between optical and electrical losses should be precisely optimized. Duong et al. optimized sputtered ITO for both the front and rear contacts, and they demonstrated a semitransparent methylammonium perovskite (MAPbI_3_) device exceeding 12% efficiency and surpassing 80% transmittance in the wavelength range of 800–1000 nm.^84^ The PCE of a perovskite–silicon multijunction device reached 20.1% when incorporating a passivated emitter rear locally diffused (PERL) silicon cell with efficiency of 19.6%. Later, Peng et al. doped TiO_2_ electron transport layers with indium to boost the fill factor (FF) and *V*_oc_ of perovskite cells and demonstrated a 16.6% efficient semitransparent perovskite cell utilizing a mixed perovskite based on MA and formamidinium (FA).^85^ Including an interdigitated back contact (IBC) silicon cell with an efficiency of 24%, the mechanically stacked multijunction cell exhibited a steady‐state efficiency of 24.5%.

Optical simulation indicated that the most efficient bandgap alignment of the subcells is 1.7 eV for the top cell and 1.1 eV for the bottom cell in a double‐junction solar cell^86^, ^87^, ^88^; whereas the most commonly used perovskite, MAPbI_3_, shows a bandgap of 1.55 eV,^89^, ^90^ which is not optimal for the perovskite–Si multijunction solar cell.^91^ Partial substitution of bromide for iodide in the MAPbI_3_ perovskite could provide a higher bandgap perovskite. However, the issue for these mixed halide MA‐based perovskites is the phase segregation under illumination,^39^, ^92^, ^93^, ^94^, ^95^ which may degrade the cell performance. It is found that replacing MA with Cs and FA can suppress phase segregation, leading to improved structural, thermal and moisture stability of perovskites.^96^, ^97^ Duong et al. introduced Rb cations into a multication perovskite and achieved a bandgap of 1.73 eV for a Rb‐FA_0.75_MA_0.15_Cs_0.1_PbI_2_Br perovskite cell with enhanced light stability.^79^ They used MoO*_x_* (10 nm)/ITO (40 nm) as the transparent electrode, which was subsequently shaded 3% by Au fingers added to compensate for the high sheet resistance of the as‐deposited ITO. The semi‐transparent perovskite cell exhibited a steady‐state efficiency of 16.0%, with a comparison opaque cell efficiency of 17.4%. It showed a very high average transparency of up to 84% in the wavelength range between 720 and 1100 nm. The IBC silicon cell with a single‐cell efficiency of 23.9% retained 10.4% under the semitransparent perovskite cell. As a result, a total efficiency of 26.4% for a mechanically stacked multijunction device was obtained, the highest efficiency for a 4T mechanically stacked perovskite–silicon multijunction solar cell so far.^79^

Another choice for the transparent contact is an ultrathin metal film formed by thermal evaporation, which is the most convenient process, and such a film does not need a buffer layer before deposition. Chen et al. employed a bilayer of Cu (1 nm)/Au (7 nm) as the transparent electrode with 22 Ω sq^−1^ sheet resistance and 51%‐64% transmittance between 800 and 1100 nm,^82^ and the semitransparent perovskite solar cell demonstrated a PCE of 16.5%. Considering the ultrathin electrode, the roughness of the underlying perovskite layer can significantly influence the electrical properties of the ultra‐thin layer; therefore, they employed a one‐step method instead of a two‐step method to synthesize the perovskite layer and obtained a smooth perovskite film. They further optimized the infrared performance of the silicon solar cell through the use of an antireflective coating. When this cell was combined with the semitransparent top cell, an overall PCE of 23% was attained.^82^ The reported 4T multijunction solar cell was composed of a small area semitransparent perovskite top cell with a large silicon bottom cell, since the tradeoff between sheet resistance and transmittance of the transparent electrode was a challenge when moving toward large‐area semitransparent cells. Jaysankar et al. proposed the module‐on‐cell concept and fabricated a 4 cm^2^ semitransparent perovskite module with an identical area IBC silicon device.^98^ The 4T perovskite‐c‐Si module exhibited an aperture‐area PCE of 20.2%. This research provides a feasible way to commercially fabricate large‐area perovskite‐c‐Si multijunction solar cells.

In the 2T monolithically integrated device, the top subcell is directly processed on the bottom subcell. As a result, only one transparent electrode is required, rather than the three transparent electrodes in a 4T multijunction device. This advantage reduces the manufacturing cost as well as the parasitic absorption loss in the transparent electrodes. However, the crucial issue is the recombination layer^52^, ^61^ or tunnel junction^55^ between two subcells. Mailoa et al. first fabricated a monolithic multijunction solar cell using perovskite and silicon devices in early 2015, and the efficiency was up to 13.7% with a *V*_oc_ of 1.56 V.^55^ They fabricated a mesoporous MAPbI_3_ perovskite cell on top of an n‐type silicon cell with Ag NWs as the transparent top electrode. To provide for carrier recombination between the two subcells, they synthesized an n^++^/p^++^ tunnel junction formed by depositing heavily doped n^++^ hydrogenated amorphous silicon (a‐Si:H) using plasma‐enhanced chemical vapor deposition (PECVD). The multijunction cell exhibited strong hysteresis, which was caused by the immature MAPbI_3_ perovskite subcell. Silicon heterojunction (SHJ) solar cells have attracted widespread attention due to their high efficiency, simple fabrication process, low temperature coefficient and high *V*_oc_.^99^, ^100^, ^101^ However, SHJ cells are unstable above 200 °C because the passivation quality of the a‐Si:H/c‐Si interface is reduced at high temperature, whereas the highest‐efficiency mesoporous perovskite used TiO_2_ sintered above 450 °C, which is unsuitable for integration with a SHJ cell. One option to solve this problem is to use a planar perovskite subcell with a compact electron transport layer (ETL) synthesized under mild conditions. Albrecht et al. reported a monolithic HIT‐perovskite multijunction solar cell with a stable power output efficiency of 18.1% in 2016.^54^ Through the utilization of atomic‐layer‐deposited SnO_2_ at low temperatures instead of the high‐temperature mesoporous TiO_2_ ETL, the perovskite subcell was fabricated on top of an SHJ cell below 100 °C. The SnO_2_ and the underlying ITO served as a recombination layer between the two subcells. Sputtered ITO was employed as the top transparent electrode, with MoO_3_ as a buffer layer and LiF as an antireflective coating.

Organic polymers provide a new choice for synthesizing an ETL at low temperature.^102^, ^103^, ^104^ Werner et al. developed a low‐temperature‐processed planar perovskite cell using a polyethylenimine (PEIE)/phenyl‐C_61_‐butyric‐acid‐methyl‐ester (PCBM) bilayer as the ETL.^52^ An MAPbI_3_ perovskite layer was formed by a two‐step deposition. First, a PbI_2_ layer was deposited onto a phenyl‐C61‐butyric acid methyl ester (PCBM) layer by thermal deposition. Then MAI, dissolved in isopropanol, was spin‐coated onto the PbI_2_ layer, followed by annealing at 100 °C for 30 min. A small amount of 2‐methyoxyethanol was added to the MAI precursor to form a homogeneous, pinhole‐free, flat perovskite layer. Sputtered IZO film was used for the recombination layers. spiro‐OMeTAD was used as the hole transport material. Hydrogenated indium oxide/indium tin oxide (IO:H/ITO) was deposited on the spiro‐OMeTAD with a MoO*_x_* buffer layer to form the transparent top electrode. When the semi‐transparent perovskite cell was fabricated on top of the silicon heterojunction cell, a steady‐state efficiency of 19.2% was achieved for the monolithically integrated multijunction solar cell with an aperture area of 1.22 cm^2^, and 21.2% was obtained with an aperture area of 0.17 cm^2^. The current was usually limited by the bottom cell in the 2T multijunction device; therefore, enhancing the infrared response of the silicon bottom cell could further improve the multijunction cell performance.

**Figure 3:**
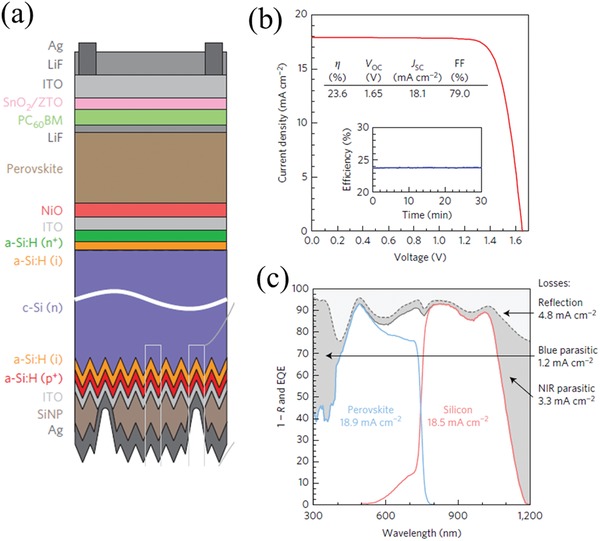
a) Schematic, b) *J–V* curve, and c) EQE of the record 2T perovskite–Si multijunction cell. Reproduced with permission.^47^ Copyright 2017, Spring Nature.

Bush et al. demonstrated 23.6% efficiency from a 2T perovskite–silicon multijunction solar cell with a 1 cm^2^ area by combining an infrared‐enhanced silicon heterojunction bottom cell with a cesium‐doped FAPbI_3_ perovskite top cell in early 2017 (**Figure** **3**).^47^ The increased moisture and thermal stability enabled the deposition of SnO_2_ by atomic layer deposition. They introduced a bilayer of SnO_2_/ZTO as the electron transport layer, which was a sufficient buffer to prevent damage to the organic and perovskite layers during the sputter‐deposition of ITO as the transparent electrode. The inverted perovskite cell structure is shown in Figure 3a. In the inverted structure, the ITO top electrode was sputtered onto the SnO_2_/ZTO bilayer without the use of a MoO*_x_* buffer layer, as MoO*_x_* was reported to react with the iodide in the perovskite/MoO*_x_* interface and raise the concern of decreasing the long term stability.^105^ The infrared photon absorption was enhanced by the random pyramids formed by the texture.^47^ The inverted structure of the perovskite could use NiO as the HTL to substitute for spiro‐OMeTAD because of its apparent absorption in the infrared region, thus reducing the parasitic absorption and leading to enhanced current.

Reducing the nonradiative recombination in perovskites is a prerequisite to obtain highest performance of solar cells, which has been proven by many works.^106^, ^107^ In a recent work by Braly and coworkers, the very high quality polycrystalline MAPbI_3_ perovskite film was fabricated and thanks to the surface passivation by n‐trioctylphosphine oxide (TOPO), the nonradiative recombination was reduced and the perovskite film exhibits very high internal photoluminescence quantum efficiencies (PLQEs) over 91%. The perovskite film showed a quasi‐Fermi‐level splitting of 1.28 eV after surface passivation, indicating that the maximum *V*_oc_ of 1.28 V could be achieved if the perovskite surface is properly passivated. The predicted PCE of the film could be improved from 24.3 to 27.9% after surface passivation by TOPO.^106^ Martin and coworkers found that interfacial recombination could result in an additional free energy loss of 80 meV at the interface between perovskite and transport layers, which limit the *V*_oc_ of the complete device to ≈1.12 V in the p–i–n type PSC. They introduced ultrathin interlayers between perovskite film and transport layers, which lead to a substantial reduction of the interfacial recombination loss at both the perovskite/ETL and perovskite/HTL interfaces. In their work, an ultrathin (0.6–1 nm) LiF layer was inserted to the perovskite/C60 interface, which reduced the interfacial recombination loss by 35 meV at the electron‐selective interface. Poly[(9,9‐bis(30‐((*N*,*N*‐dimethyl)‐*N*‐ethylammonium)‐propyl)‐2,7‐fluorene)‐*alt*‐2,7‐(9,9‐dioctylfluorene)] dibromide (PFN‐P2) was used to passivate the perovskite/PTAA interface, and the recombination at the hole transport later contact was substantial reduced. Thus they have obtained 20.0% efficiency in 1 cm^2^ device with an improved *V*_oc_ of 1.17 V.^107^

To further increase the performance of perovskite‐based multijunction device, it is great importance to improve the quality of perovskite. Many works have been done to improve the perovskite itself, such as adding multiple cations or halides into the lattice,^43^ or including additives into the perovskite precursor solution.^108^, ^109^ In a recent work by Huang group, grain engineering was applied to improve the perovskite performance. They utilized MACl and MAH_2_PO_2_ as additives to the perovskite precursor solution. MACl enlarges the grain size, and MAH_2_PO_2_ reduces nonradiative recombination since it can passivate perovskite grain boundary. The *V*_oc_ deficit of the wide‐bandgap perovskite solar cells are reduced to 0.49–0.51 V, and the resulting perovskite/silicon monolithic tandem device showed a high *V*_oc_ of 1.80 eV and improved PCE of 25.4%. However, the front side of the silicon cell was polished, which increased reflection loss and limit the *J*_sc_ to a junior level of only 17.8 mA cm^−2^.^110^ Sahli et al. demonstrate a fully textured monolithic perovskite/silicon tandem solar cell with a PCE of 25.2%. Compared with the perovskite/silicon tandem device with front‐side‐polished (FSP) bottom cells, the tandem device with double‐side‐textured (DST) bottom cell exhibits reduced reflection loss of 3.14–1.64 mA cm^−2^. The optimal tandem device showed an enhanced *J*_sc_ of 19.5 mA cm^−2^.^111^ Meanwhile, Oxford PV demonstrated the 27.3% PCE 1 cm^2^ perovskite/silicon tandem solar cell on 2018 June 25, which has been certified by the Fraunhofer Institute for Solar Energy System ISE. It first time exceeds the 26.7% efficiency world record for a single‐junction silicon solar cell, and has been the world record double‐junction perovskite/silicon monolithic tandem solar cell.^112^

Uzu et al. incorporated an optical splitter into the multijunction architecture and produced an optically coupled perovskite‐SHJ 4T multijunction solar cell with efficiency of 28% in late 2014.^113^ In their device, an optical splitter was positioned 45° with respect to each subcell, and the solar radiation was split by a 550 nm cutoff splitter. As a result, the shorter wavelength photons were reflected to the wide bandgap MAPbI_3_ perovskite top cell with an efficiency that varied from an original unsplit value of 15.3% to 7.5% in the split configuration, while the longer wavelength photons were transmitted to the narrow bandgap SHJ bottom cell with an efficiency that varied from 24.1% unsplit to 19.9% split. They found that increasing the cutoff wavelength of the splitter could lead to a reduced total efficiency, which was caused by the smaller external quantum efficiency (EQE) of the perovskite top cell compared with that of the SHJ bottom cell at λ > 550 nm. Considering that only the light below the cutoff wavelength contributes to the current of the top MAPbI_3_ perovskite cell, they suggested that a wider bandgap cell with a higher *V*_oc_ than MAPbI_3_ could maintain the current when the 550 nm cutoff splitter was used, and thus the total efficiency could be enhanced.^113^ Lately, Sheng et al. used bromide to substitute for iodide and fabricated a higher bandgap (2.3 eV) MAPbBr_3_ perovskite cell as the top cell in an optically coupled multijunction device.^114^ Coupled with a 22.7% efficient PERL silicon solar cell, the total efficiency reached 23.4%, slightly higher than that of the single PERL cell. The slight improvement was mainly caused by the low EQE (40%–50%) at λ < 550 nm and high *E*_g_/*e*–*V*_oc_ offset for the MAPbBr_3_ perovskite cell.^51^, ^114^ Therefore, developing an efficient high‐bandgap perovskite cell with high EQE and a small *E*_g_/*e*–*V*_oc_ offset can further enhance the efficiency of the optically coupled multijunction solar cell. In addition, considering that the bandgap of 2.3 eV is not optimal for the top cell of a silicon‐based multijunction solar cell, a well‐managed light splitter is essential for improving the efficiency.

### 3.2 Perovskite–CIGS Multijunction Solar Cells

Both perovskite and CIGS are thin film technologies, and can be fabricated on flexible substrates in a cost‐effective manner. In addition, the bandgaps of perovskite and CIGS in combination would result in widened absorption of the solar spectrum. Therefore, integrated photovoltaics and mobile devices using perovskite and CIGS have enormous potential.

Todorvo et al. demonstrated a perovskite–CIGS monolithic multijunction solar cell in 2015 with the structure: transparent conducting electrode (TCE)/ PCBM/perovskite/poly(3,4‐ethylenedioxythiophene) polystyrene sulfonate (PEDOT:PSS)/ITO/CdS/CIGS/MoSi_3_N_4_/glass. Because ZnO in proximity to the perovskite layer could degrade the device performance, the 30 nm thick ITO recombination layer was directly deposited onto CdS without the commonly used intrinsic ZnO in the CIGS cell.^56^ They invented a bandgap engineering method that can tune the bandgap of MAPb(I*_x_*Br_1‐_ *_x_*)_3_ perovskite reversibly and continuously. They synthesized a bandgap of 1.04 eV for the CIGS cell and varied the bandgap of perovskite from 1.65 to 1.75 eV. The highest‐efficiency multijunction device with a Ca (10–15 nm)/bathocuproine (BCP) (≈5 nm) transparent electrode reached an efficiency of 10.98% using a perovskite layer with a bandgap of 1.72 eV, where the BCP was used as an n‐selective layer and Ca was used as a diffusion barrier. The relatively low PCE was limited by the high parasitic absorption of the transparent electrode. When a superior transparent electrode is employed, the cell performance will be enhanced.

Bailie et al. reported an 18.6%‐efficient 4T perovskite/CIGS multijunction cell. As we discussed above, mechanically transferred Ag NWs were used as the top transparent electrode of the perovskite cell with transmission above 87% in the wavelength range from 600 to 1000 nm.^70^ Similar to the perovskite/Si multijunction cell, TCOs and ultrathin metals have also been used as the transparent electrode of perovskite/CIGS multijunction cells. Kranz et al. demonstrated a 4T perovskite/CIGS multijunction cell with AZO/MoO*_x_* as the transparent electrode.^74^ The efficiency of the semitransparent top cell reached 12.1% with an average sub‐bandgap transmission of 71% in the wavelength range from 800 to 1000 nm. The original 18.4%‐efficiency CIGS cell retained 7.4%‐efficiency when filtered by the semitransparent perovskite top cell. A total efficiency of 19.5% was achieved for the 4T multijunction cell.

Guchhait et al. compared Ag and MoO*_x_* as the buffer layer for ITO deposition in the top electrode.^115^ They found a significant drop in the FF when MoO*_x_* was used as the buffer layer instead of Ag. The decreased FF may be caused by the poor vertical conductivity/recombination defects in MoO*_x_*. An efficiency of 16% was obtained for a triple‐cation semitransparent perovskite cell along with more than 50% transparency in the near‐infrared region. When the perovskite cell was combined with a 12.3%‐efficiency CIGS cell, the efficiency of the 4T perovskite/CIGS solar cell reached 20.7%.

Recently, Shen et al. demonstrated 23.9%‐efficiency for a mechanically stacked perovskite/CIGS multijunction solar cell, which exceeded the record single‐junction CIGS solar cell efficiency of 22.6%.^116^ They adopted IZO (40 nm) as the transparent electrode of the perovskite, with MoO*_x_* as the buffer layer and a metal grid to enhance the conductivity. The efficient and stable wide‐bandgap Cs_0.05_Rb_0.05_FA_0.765_MA_0.135_I_2.55_Br_0.45_ semitransparent perovskite cell with excellent transparency between 700 and 800 nm had been the key factor enabling the high efficiency multijunction solar cell.

### 3.3 Perovskite–Perovskite Multijunction Solar Cells

Perovskite–perovskite multijunction solar cells provide a full‐solution process to fabricate multijunction cells. To fabricate an efficient all‐perovskite multijunction solar cell, the topmost issue is to develop both wide‐bandgap and narrow‐bandgap perovskite cells with efficient performance. The highest‐efficiency double‐junction cell requires a top cell with a bandgap of 1.7–1.9 eV and a bottom cell with a bandgap of 0.9–1.2 eV.^91^

Chen et al. first proposed the all‐perovskite‐based multijunction cell in 2014.^117^ They studied the optical properties by Variable‐angle spectroscopic ellipsometry (VASE) and simulated the optimum‐performance single‐ and multijunction planar‐type perovskite cell. MAPbI_3_ was assumed as the active layer for both the top and bottom cell. The calculated maximum efficiency of the multijunction cell was approximately equal to that of the single‐junction cell because the high‐extinction‐coefficient perovskite layer has the potential to absorb most of the photons in a single layer, whereas the multijunction cell achieved a high *V*_oc_ of 2.2 V, leading to application in water splitting.^118^, ^119^ Later, Heo et al. provided a novel method to fabricate a perovskite–perovskite 2T multijunction solar cell by simply laminating a single‐junction MAPbBr_3_ cell with a single‐junction MAPbI_3_ cell.^120^ For the MAPbBr_3_‐MAPbI_3_ multijunction cell, they simply laminated the FTO/bl‐TiO_2_/MAPbBr_3_/wet P3HT or PTAA substrate and the PCBM/MAPbI_3_/PEDOT: PSS/ITO substrate by pressurizing with a double clip and substrate drying. The efficiency of the multijunction cell reached 10.4% with a *V*_oc_ of 2.25 V and a short‐circuit current density (*J*_sc_) of 8.3 mA cm^−2^. This technology provides a useful method for fabricating perovskite‐based 2T multijunction solar cells.

**Figure 4:**
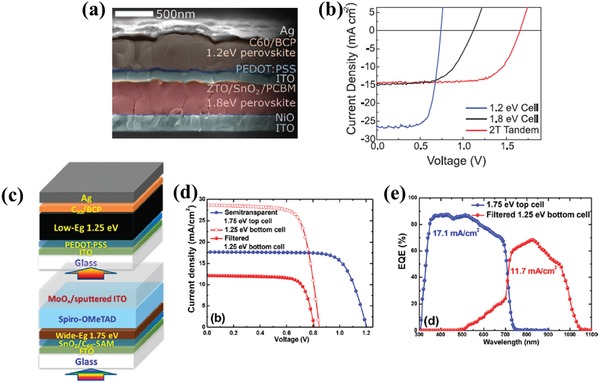
Perovskite–perovskite multijunction solar cells. a) Cross‐sectional SEM and b) *J–V* curve of the 2T multijunction cell with a 1.2 eV bandgap bottom cell and a 1.8 eV bandgap top cell reproduced from Eperon et al. Reproduced with permission.^46^ Copyright 2016, AAAs. c) Illustration, d) *J–V* curve, and e) EQE of the 4T all‐perovskite multijunction cell. Reproduced with permission.^64^ Copyright 2018, American Chemical Society.

Eperon et al. demonstrated a stable 14.8%‐efficient perovskite solar cell based on a 1.2 eV bandgap FA_0.75_Cs_0.25_Pb_0.5_Sn_0.5_I_3_ absorber. Combined with the 1.8 eV bandgap FA_0.83_Cs_0.17_Pb(I_0.5_Br_0.5_)_3_ perovskite cell, they obtained a 17%‐efficient 2T multijunction cell (**Figure** **4**a,b).^46^ They also demonstrated a 20.3%‐efficient all‐perovskite 4T multijunction cell using a 1.6 eV bandgap FA_0.83_Cs_0.17_Pb(I_0.83_Br_0.17_)_3_ semitransparent top cell. Zhao et al. reported an all‐perovskite 4T multijunction solar cell with an efficiency of 21.0% in early 2017.^63^ They synthesized an efficient mixed tin‐lead iodide perovskite with a low bandgap of 1.25 eV and efficiency of 17.6%. When combined with the 1.58 eV bandgap top cell, with MoO*_x_*/Au/MoO*_x_* as the transparent electrode, the low‐bandgap perovskite bottom cell retained only 3% efficiency, which was limited by the lower transmission of the MoO*_x_*/Au/MoO*_x_* electrode and the relatively low perovskite bandgap of the 1.58 eV top cell compared with the optimized 1.7 eV bandgap for the top cell in the two‐junction multijunction solar cell. To further enhance the efficiency of the all‐perovskite multijunction cell, a top cell with 1.7 eV bandgap perovskite is crucial. Recently, a 4T perovskite–perovskite multijunction solar cell with efficiency above 23% was demonstrated (Figure 4c‐e).^64^ That is the first reported all‐perovskite multijunction solar cell to have a PCE higher than the world record PCE for a single‐junction perovskite solar cell. Instead of the 1.58 eV bandgap of the FA_0.3_MA_0.7_PbI_3_ perovskite absorber, they developed an efficient semitransparent 1.75 eV wide‐bandgap FA_0.8_Cs_0.2_Pb(I_0.7_Br_0.3_)_3_ perovskite top cell. They substituted MoO*_x_*/ITO for MoO*_x_*/Au/MoO*_x_* as the transparent electrode, which insured more photons reached the low‐bandgap cell. The wide‐bandgap semitransparent perovskite cell showed an average transmittance of ≈68% between 700 and 1100 nm. What is more, they applied paraffin oil as an optical coupling spacer between the two subcells, thus reducing the optical loss of the wide‐bandgap bottom cell caused by reflection at the surface of the bottom subcell. As a result, the filtered low‐bandgap perovskite bottom cell retained 7.4% efficiency, which was much higher than in their previous work.^63^

### 3.4 Perovskite–Polymer Multijunction Solar Cells

Polymer solar cells share similar processing and architecture characteristics with perovskite cells. Since polymer cells with excellent near‐IR response have been reported,^121^, ^122^ it is meaningful to integrate polymer cells with perovskite cells to form perovskite–polymer multijunction solar cells. However, if a perovskite cell were to be fabricated directly on top of a polymer cell, the annealing process for the perovskite would damage the polymer subcell.

Chen et al. demonstrated a perovskite–polymer multijunction solar cell toward the end of 2014.^60^ They used an additive‐assisted solvent wash method to control the crystal growth process, and the small‐molecule additive BmPyPhB served as a heterogeneous nucleation site source to form a continuous and dense film without large voids. The MAPbI_3_ single‐junction solar cell attained 9.1% efficiency when formed by annealing at 100 °C for 5 min, which was suitable for fabrication on the polymer solar cell. They introduced a new IR‐polymer, PBSeDTEG8 with excellent thermal stability as the bottom cell, which could withstand the treatment process of the top perovskite film. The efficiency of the monolithically integrated perovskite–polymer multijunction cell reached 10.23%.

Liu et al. fabricated a polymer cell on top of a perovskite subcell, thus avoiding the polymer degradation during the perovskite thermal annealing process.^61^ They fabricated a very thin perovskite layer (≈90 nm) to ensure enough photons would be absorbed by the polymer. They obtained a total efficiency of 16.0% for the perovskite–polymer multijunction solar cell with a high *V*_oc_ of 1.80 V. Development of technologies for fabricating perovskite cells using a low temperature annealing process could mitigate the effect on polymer cells, thus leading to superior‐performance perovskite–polymer multijunction solar cells.

## 4 Prediction of the Efficiency and Cost for a Perovskite–Silicon Double‐Junction Cell

Recently, perovskite–silicon double‐junction solar cells have been researched by many groups, and the efficiency has been increased from 13.7% to 27.3%.^55^, ^79^ In the past few years, the PCE of single‐junction perovskite solar cells has reached 22.7%. The quickly increasing efficiency of single‐junction perovskite cells could promote the development of perovskite‐based double‐junction cells. By assuming optimization of the film thickness of the multilayers, utilization of low parasitic absorption charge transport materials with high charge extraction ability, and the design of light trapping structures by surface texturing, we can give an outlook for the efficiency of perovskite–silicon double‐junction solar cells. If MAPbI_3_ was used as the perovskite absorber, we could obtain a *V*_oc_ of ≈1.2 V and an FF of ≈0.80 for the perovskite top‐cell. As to the silicon partner, we assumed a PCE of 25% with *V*_oc_ of 0.75 V and FF of 0.8. By plotting the detailed balance limit and optimizing the parameters of the double‐junction model, we obtain a *J*_sc_ of 22.0 mA cm^−2^ for the perovskite top‐cell and 16.0 mA cm^−2^ for the silicon bottom cell under AM 1.5G conditions with a reflection loss of 3.6 mA cm^−2^. As a result, efficiencies of the top cell and bottom cell are 21.1% and 9.6%, respectively, and the total efficiency of the double‐junction device reaches 30.7%.

Making solar cells competitive with energy from traditional fossil fuel sources requires lower cost compared with other energy sources. Since PV system cost scales with the installation area, and thus inversely with the module efficiency, improving the module efficiency by using double‐junction cells has been a promising way to reduce the system cost. The module cost can be divided into the capital cost, material cost and overhead cost. Cai et al. estimated the module cost of perovskite solar cells to be 0.25 dollar Watt^−1^ as the sum of the capital cost, material cost and overhead cost, which is one third of the module cost of bulk silicon solar cells.^123^ A perovskite–silicon double‐junction module commonly requires 1% additional capital cost relative to a single‐junction silicon module, and the increased material cost of a perovskite–silicon double‐junction module can be neglected owing to the very cheap perovskite materials. Considering the high predicted efficiency (30.7%) of the double‐junction device, we therefore can expect that integrating perovskite into the dominant silicon module could effectively reduce the PV system cost.

## 5 Design of Perovskite‐Based Triple‐Junction Solar Cell

To the best of our knowledge, no perovskite‐based triple‐junction solar cells have been reported. However, triple‐junction polymer solar cells have been demonstrated with efficiency over 11%.^124^ Since perovskite solar cells share a similar structure with polymer solar cells and a few triple‐junction polymer cells have been demonstrated, it is likely that a perovskite triple‐junction solar cell will be designed. Compared to the double‐junction device, a triple‐junction device possesses a higher power conversion efficiency limit due to the reduced thermalization loss of hot excitons and enhanced photon absorption in the long wavelength region. In accordance with Kirchhoff's laws, triple‐junction devices could produce very high *V*_oc_. For example, Chen demonstrated a triple‐junction polymer solar cell comprised of P3HT:ICBA (1.9 eV), PTB:PC_71_BM (1.58 eV) and LBG:PC_71_BM absorbers that produced an overall *V*_oc_ of 2.28 V.^124^ Regarding a perovskite‐based triple‐junction device, the bandgap of the three subcells should be 0.9–1.2, 1.5–1.7, and 1.9–2.1 eV, respectively.^125^ Hörantner et al. used a transfer matrix to simulate a more realistic expectation for the PCE of a perovskite‐based triple‐junction solar cell. They simulated an all‐perovskite triple‐junction device and a perovskite–perovskite–silicon triple‐junction device by varying the bandgaps of the top cell and middle cell while fixing the bandgap at 1.22 eV for the perovskite bottom cell and at 1.1 eV for the silicon bottom cell. The all‐perovskite triple‐junction device showed a slightly higher efficiency than an all‐perovskite double‐junction device (from 32.2% to 33%) with a very high *V*_oc_ of 3.5 eV. However, the perovskite–perovskite–silicon triple‐junction devices exhibited an efficiency of 35.3%, which is much higher than the 31.8% of a perovskite–silicon double‐junction device.^125^ Those results suggest that developing a perovskite–perovskite–silicon triple‐junction device may be a promising way to further enhance efficiency.

## 6 Perspective of Perovskite‐Based Solar Cells in Portable Electronic Device

Portable devices have been attracting a considerable amount of attention since Google Inc. launched Google Glass in 2012. Up to now, portable devices have played an important role in the consumer electronic market. However, the most important issue of portable devices is the energy source. The high‐efficient tandem solar cells potentially enable the portable device to work continuously even if there are no batteries or only small capacity batteries are required as the backup power. Perovskite‐based solar cells exhibit excellent dim‐light performance, and this advantage makes PSCs the promising power source for portable devices under indoor condition.^126^

Typically, the household lighting ranges from 100 to 1000 lux. These illuminances correspond to 31–310 µW cm^−2^ light intensity with a 6500 K fluorescent lamp spectrum. In general, both fluorescent and light‐emitting diode (LED) lamps exhibit illumination wavelength ranges from 350 to 750 nm, and ≈1.6 eV bandgap of perovskite materials cover the whole spectra. Therefore, when the perovskite cell works under indoor condition, the energy loss caused by photon loss in the infrared range would be avoided. Thus perovskite cells could perform more efficient under indoor condition compared to sunlight condition if they can maintain a high *V*_oc_ and FF under dim‐light illumination. Actually, some reports have shown above 27%‐efficiency under indoor light for single‐junction perovskite solar cells.^126^, ^127^ In the multijunction perovskite tandem solar cell, the PCE could be higher. Assuming 35%‐efficiency could be achieved for a multijunction cell, it means that 100 mW power output requires less than 1000 cm^2^, which is available in a bag. This energy is enough to power some wireless sensors, portable electronics and Internet of Things. Amazingly, it could by fabricated to some portable devices thanks to the softness of perovskite materials.

**Figure 5:**
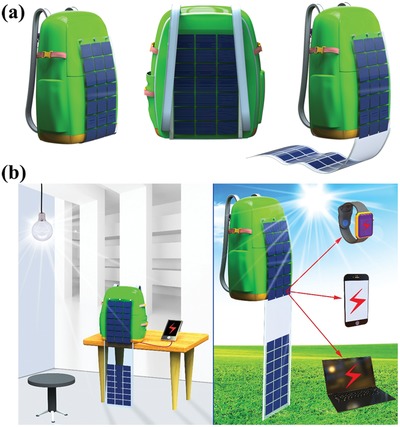
Illustration of perovskite‐based solar cell integrates with some portable devices. a) Bag with both sides covered by perovskite‐based solar cell. b) Cases of indoor and outdoor situation for the perovskite‐based solar cell covered bag to charge the smart phones, smart watches and laptop.

Here we demonstrate some cases that perovskite‐based solar cells applied in portable electronic devices, as shown in **Figure** **5**. A bag could be covered by solar cells for both sides to enlarge illumination area and generate a higher power. As a result, it could not only be the power source for smart phones even under ambient light indoors, but also to power the laptop outdoors.

## 7 Conclusion

OIHPs have been proven to be excellent top cell materials for multijunction solar cells due to their tunable bandgap, easy fabrication and low cost. The record highest‐efficiency perovskite–silicon multijunction solar cell has reached an efficiency of 27.3% and is predicted to achieve 30.7% in the near future. Since c‐Si solar cells are the dominant technology in the current PV market, incorporating perovskite cells into commercial c‐Si solar cells may provide a feasible path to commercialization of perovskite solar cells. Considering that all of the CIGS solar cells, polymer solar cells and perovskite solar cells can be fabricated on flexible substrate, multijunction solar cells based on these technologies could be used in portable devices. The multijunction cells could be used in some particular applications, such as water splitting or with secondary batteries, due to their very high *V*_oc_. Further improvement of the performance of multijunction cells involves some issues: i) The lack of an efficient wide‐bandgap perovskite with a high *V*_oc_. ii) The reduction of *V*_oc_ deficit in wide‐bandgap PSCs. Since the nonradiative recombination at the interface between perovskite and transport layers as well as the boundaries of perovskite grains could leads to reduced *V*_oc_, the passivation of the interface and grain boundaries could improve the photovoltaic performance remarkably. iii) A superior method is needed for fabricating large‐area perovskite while retaining high efficiency. Compositional engineering provides a convenient method for tuning the bandgap of perovskite continuously, and the wide‐bandgap and low‐bandgap perovskites required by multijunction devices have been synthesized. However, the stability of those wide‐ and low‐bandgap materials is still insufficient for meeting the requirements of multijunction cells. To enable realistic application of perovskite‐based multijunction cells, the stability of perovskite cells needs to be comparable to those of other relevant technologies, such as c‐Si or CIGS. Moreover, the reported single‐junction perovskite cells and multijunction cells have been fabricated in small areas. Developing large‐area perovskite cell with high efficiency and superior stability may help the perovskite technology achieve commercialization.

## Conflict of Interest

The authors declare no conflict of interest.
